# Recurrent Acute Coronary Syndrome in Young Man with Familial Hypercholesterolemia: Efficacy of Evolocumab Add-On Treatment Demonstrated via Serial Coronary Angiography

**DOI:** 10.3390/biomedicines12051113

**Published:** 2024-05-17

**Authors:** Narae Kim, Jin-Man Cho, In-Ho Yang

**Affiliations:** Department of Cardiology, Kyung Hee University Hospital at Gangdong, College of Medicine, Kyung Hee University, Seoul 05278, Republic of Korea; knr802@naver.com (N.K.); cardiocho@gmail.com (J.-M.C.)

**Keywords:** acute coronary syndrome, lipid-lowering therapy, evolocumab, coronary angiography, optical coherence tomography

## Abstract

In patients with acute coronary syndrome (ACS), lipid-lowering therapy plays an important role in the prevention of the recurrence of cardiovascular disease. Recent guidelines recommend the use of proprotein convertase subtilisin/kexin type 9 (PCSK9) inhibitors in patients with ACS if their low-density lipoprotein cholesterol (LDL-C) levels are not adequately controlled with statins and ezetimibe. Based on this, we report a case in which administering a PCSK9 inhibitor successfully lowered the patient’s LDL-C level to the target level and managed the coronary artery disease (CAD) recurrence. A 39-year-old man who was taking statins presented to the hospital with chest pain and was diagnosed with unstable angina. He started taking maximum doses of statins and ezetimibe to lower his LDL-C. However, the patient’s unstable angina recurred 1 year later, and a de novo lesion with plaque rupture was demonstrated via coronary angiography. The LDL-C failed to reach the target level despite maintaining the maximum dose of statin and ezetimibe. Accordingly, evolocumab was initiated in addition to rosuvastatin/ezetimibe 20/10 mg daily. Subsequently, coronary angiography was performed twice, and on follow-up, the patient remained free of CAD recurrence. This case highlights the efficacy of lipid-lowering therapy with evolocumab in high-risk patients with repeated ACS.

## 1. Introduction

According to recent guidelines, lipid-lowering therapy is considered important in patients with acute coronary syndrome (ACS) [1,2]. The target low-density lipoprotein cholesterol (LDL-C) level for patients with ACS is below 55 mg/dL [1,2]. A proprotein convertase subtilisin/kexin type 9 (PCSK9) inhibitor is recommended if patients are unable to reach the LDL-C target with statins and ezetimibe [1,2]. However, data on the efficacy and safety of PCSK9 inhibitors in the acute phase in patients with ACS are scarce. Although one study demonstrated that evolocumab in combination with a statin effectively and safely reduced LDL-C levels over the first 8 weeks in patients in the acute phase (within 3 days) of ACS, long-term data are still lacking [3]. According to a recent study using long-term data, patients using evolocumab for 5 years experienced no serious side effects [4]. Therefore, long-term use of PCSK9 inhibitors from the acute phase of ACS is expected to be beneficial, but existing data are insufficient. Using this case as an example, we would like to report the effect of lipid-lowering therapy using evolocumab observed through serial coronary angiography in a patient with recurrent ACS.

## 2. Case Description

**Figure 1:**
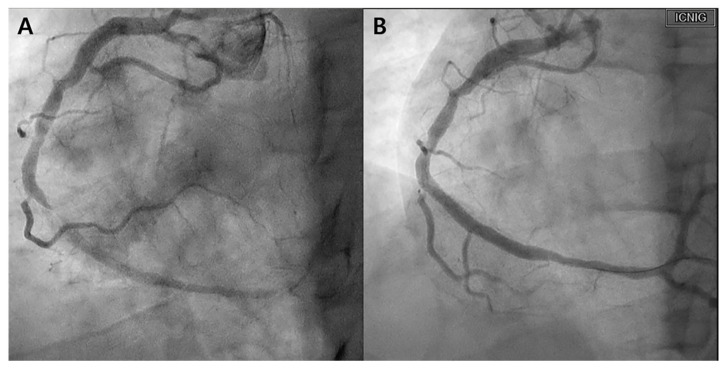
Initial coronary angiography. (**A**) Right coronary angiography performed before the intervention. Subtotal occlusion with flow limitation is observed in the distal right coronary artery (RCA). (**B**) Right coronary angiography post-intervention. After stent insertion, the RCA flow recovered.

A 39-year-old man with hypercholesterolemia presented with chest pain. He had been experiencing exertional angina for 2 months, with symptoms worsening over the past 2 weeks. Atorvastatin 20 mg (Atrovan, KyungDong Pharm., Seoul, Republic of Korea) daily was prescribed for hypercholesterolemia. Upon hospitalization for suspected unstable angina, the patient underwent echocardiography and coronary angiography. Although echocardiography yielded unremarkable findings, coronary angiography revealed prominent stenosis in the distal right coronary artery (RCA), necessitating revascularization in the form of intracoronary stent implantation (Figure 1).

Despite atorvastatin therapy, blood tests indicated uncontrolled LDL-C levels (triglyceride (TG), 133 mg/dL; high-density lipoprotein cholesterol (HDL-C), 41 mg/dL; LDL-C, 214 mg/dL). Subsequently, the patient’s medication was adjusted to atorvastatin/ezetimibe 40/10 mg (Atozet, MSD Korea Co., Rahway, NJ, USA) daily.

**Figure 2:**
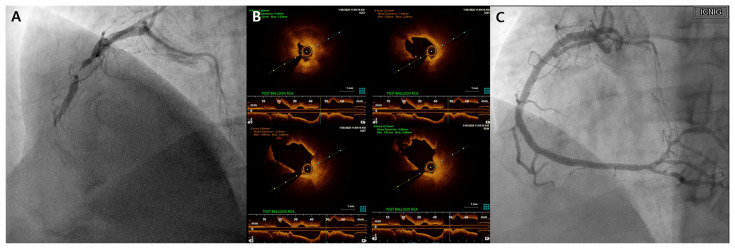
Second coronary angiography. (**A**) Right coronary angiography before intervention. Subtotal occlusion has newly developed in the proximal right coronary artery (RCA), outside the proximal part of the previous stent. (**B**) Optical coherence tomography. Plaque rupture is observed in the de novo lesions of the proximal RCA. (**C**) Right coronary angiography post-intervention. The flow recovered after stent insertion into the proximal RCA.

One year later, the patient presented again with complaints of chest pain and shortness of breath during exercise. Subsequent coronary angiography revealed a newly developed subtotal occlusion causing TIMI grade 1 flow in the proximal RCA area (Figure 2A). Optical coherence tomography (OCT) was then performed to identify the lesion features, revealing a de novo lesion accompanied by plaque rupture (Figure 2B). A new stent was promptly inserted at the lesion site (Figure 2C).

Despite being on maximum doses of statin/ezetimibe therapy, the patient’s LDL-C level remained uncontrolled (TG, 113 mg/dL; HDL-C, 38 mg/dL; LDL-C, 127 mg/dL). Genetic testing confirmed familial hypercholesterolemia, revealing a heterozygous mutation in the LDL-receptor gene. Consequently, a decision was made to administer evolocumab, a PCSK9 inhibitor, aiming to prevent future ACS recurrence by controlling LDL-C. The patient received 140 mg of subcutaneous evolocumab injections once every 3 weeks in addition to rosuvastatin/ezetimibe 20/10 mg (Crezet, Daewoong Pharm., Seoul, Republic of Korea). Subsequently, the patient’s LDL-C level consistently remained at <55 mg/dL.

**Figure 3:**
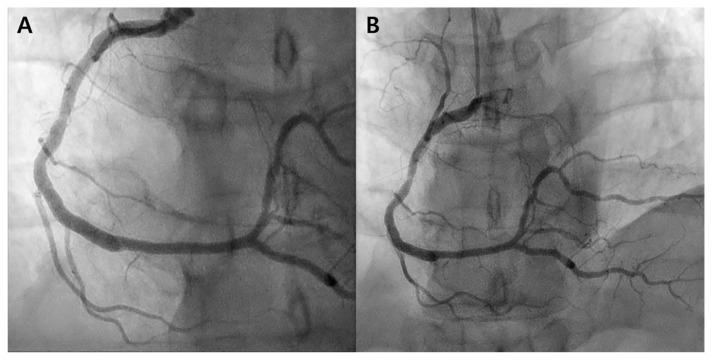
Follow-up coronary angiography. (**A**) Right coronary angiography 1 year after evolocumab administration reveals patient stents with no evidence of de novo lesions. (**B**) Right coronary angiography performed 3 years after evolocumab administration displays no in-stent stenosis or de novo lesions.

Coronary angiography was performed 1 and 3 years after evolocumab treatment, revealing no target lesion failure or de novo lesions (Figure 3). Presently, the patient maintains medication, is undergoing outpatient follow-up, and is free of specific symptoms.

## 3. Discussion

In cases of ACS recurrence within 1 year, the likelihood of stent thrombosis is notably high, and uncommon due to the rapid progression of atherosclerosis. However, in this case, ACS stemmed from atherosclerosis progression and plaque rupture, attributable to uncontrolled hypercholesterolemia despite maximum doses of statin and ezetimibe. Current guidelines underscore the significance of lipid-lowering therapy in ACS, advocating for a target LDL-C level of 55 mg/dL for patients with ACS [1,2]. The use of a PCSK9 inhibitor is recommended when patients fail to attain the LDL-C target with statins and ezetimibe [1,2].

Although the precise efficacy of PCSK9 inhibitors in the acute phase of ACS remains unclear, several studies have supported their advantages. ODYSSEY OUTCOME and FOURIER trials demonstrated that when patients with ACS were followed up on over 3 years, the likelihood of recurrent coronary artery disease (CAD) significantly decreased with the concurrent use of alirocumab or evolocumab alongside statins [5,6]. Despite limited data on the prolonged use of PCSK9 inhibitors, findings from the FOURIER-OLE trial, which followed the evolocumab treatment group for up to 5 years, indicated that the reduction in LDL-C achieved through evolocumab correlated with a sustained decline in CAD risk for >8 years, without notable side effects [4].

PCSK9 protein prevents the lowering of LDL-C levels by inhibiting LDL receptor-mediated LDL removal. PCSK9 inhibitors work by binding to PCSK9 protein and blocking its action, thereby lowering LDL-C levels [7,8,9]. According to previous studies, patients with familial hypercholesterolemia, associated with several genetic mutations, have elevated expression levels of PCSK9 protein [8]. The use of PCSK9 inhibitors is recommended to control LDL-C levels in these high-risk patients [9]. Although the mechanism by which PSCK9 inhibitors effectively prevent ACS remains unclear, research highlights their efficacy. Through this efficacy, lowering LDL-C levels by administering evolocumab may effectively prevent ACS by reducing plaque burden and stabilizing plaque phenotype [10]. Studies have demonstrated that combining evolocumab with statins post-ACS is more effective in stabilizing and effecting regression of plaques than statins alone [10]. A recent study demonstrates that alirocumab treatment is related to plaque burden regression, similar to that induced by evolocumab [11]. These findings have led both the American College of Cardiology/American Heart Association and the European Society of Cardiology guidelines to recommend LDL-C level reduction through medications, including PSCK9 inhibitors, for patients with ACS.

## 4. Conclusions

Here, we present a case involving the treatment of recurrent ACS with evolocumab in a patient with familial hypercholesterolemia. This case illustrates the recurrence of ACS episodes characterized by plaque rupture in a patient whose LDL-C levels were insufficiently controlled with statins and ezetimibe. The significance of this case lies in our serial observation of the management of CAD through evolocumab administration in such patients. Additionally, the case highlights the critical importance of rigorous lipid-lowering therapy, particularly employing evolocumab, in high-risk patients. Evolocumab should be used aggressively in patients who do not respond to statin therapy.

## Data Availability

The data presented in this study are available on request from the corresponding author.
